# Freeze–Thaw Damage Characteristics of Concrete Based on Compressive Mechanical Properties and Acoustic Parameters

**DOI:** 10.3390/ma17051010

**Published:** 2024-02-22

**Authors:** Dongye Lv, Hanbing Liu, Feng He, Wensheng Wang, Qiang Miao, Hanjun Li, Fuen Wang, Jing Zhao, Chengwei Shi

**Affiliations:** 1 College of Transportation, Jilin University, Changchun 130025, China; lvdy21@outlook.com (D.L.); liuhb-jlu@outlook.com (H.L.); miaoq21@outlook.com (Q.M.); 2 Jilin China Railway Expressway Co., Ltd., Changchun 130052, China; lihanjun-crecg@outlook.com (H.L.); wangfuen@crecg.com (F.W.); 18813098377@163.com (J.Z.); 3 Jilin Traffic Planning and Design Institute, Changchun 130021, China; shicw80@outlook.com

**Keywords:** concrete, freeze–thaw, compressive strength, ultrasonic, acoustic emission

## Abstract

Concrete is a versatile material widely used in modern construction. However, concrete is also subject to freeze–thaw damage, which can significantly reduce its mechanical properties and lead to premature failure. Therefore, the objective of this study was to assess the laboratory performance and freeze–thaw damage characteristics of a common mix proportion of concrete based on compressive mechanical tests and acoustic technologies. Freeze–thaw damage characteristics of the concrete were evaluated via compressive mechanical testing, mass loss analysis, and ultrasonic pulse velocity testing. Acoustic emission (AE) technology was utilized to assess the damage development status of the concrete. The outcomes indicated that the relationships between cumulative mass loss, compressive strength, and ultrasonic wave velocity and freeze–thaw cycles during the freezing–thawing process follow a parabola fitting pattern. As the freeze–thaw damage degree increased, the surface presented a trend of “smooth intact surface” to “surface with dense pores” to “cement mortar peeling” to “coarse aggregates exposed on a large area”. Therefore, there was a rapid decrease in the mass loss after a certain number of freeze–thaw cycles. According to the three stages divided by the stress–AE parameter curve, the linear growth stage shortens, the damage accumulation stage increases, and the failure stage appears earlier with the increase in freeze–thaw cycles. In conclusion, the application of a comprehensive understanding of freeze–thaw damage characteristics of concrete based on compressive properties and acoustic parameters would enhance the evaluation of the performance degradation and damage status for concrete structures.

## 1. Introduction

Concrete is a widely selected construction material because of its higher compressive strength, excellent durability, and ease of use [1,2,3,4]. Nevertheless, concrete is also subject to freeze–thaw damage, which can significantly reduce its mechanical properties and lead to premature failure [5]. Concrete construction in cold climates presents several other material-related challenges such as slower strength development, the risk of early freezing, and thermal stresses due to internal temperature differences [6,7,8]. Therefore, understanding the freeze–thaw damage characteristics of concrete is crucial for ensuring its performance and durability.

Freeze–thaw cycles change the physical and mechanical properties of materials [9,10,11]. In recent years, researchers have conducted numerous studies on the freeze–thaw damage characteristics of concrete. These studies have focused on various aspects, including the relationship between mechanical properties and freeze–thaw damage, the mechanisms responsible for freeze–thaw damage [12,13], and methods for enhancing anti-freeze thawing performance [14,15].

One line of research has investigated the relationship between mechanical properties and freeze–thaw damage in concrete [16]. Previous studies have shown that the compressive strength, elastic modulus, and other properties of concrete decrease with freezing–thawing [17]. Gong et al. discussed different stages of specimens under uniaxial compression and their performances after being damaged, and they pointed out that there is a significant linear relationship between mechanical parameters during uniaxial compression and freeze–thaw damage [18]. In addition, in view of the strength attenuation during freezing–thawing, many scholars have tried to analyze the causes of strength weakening in the micro-mechanism of concretes. Zahedi et al. evaluated the damage of concrete specimens after freeze–thaw cycles by combining mechanical and microscopic results in Canada and the United States for a global assessment [19]. Common test methods such as scanning electron microscopy, nitrogen adsorption, and nuclear magnetic resonance testing are gradually being used to analyze the micro-characteristics of concrete materials and structures [20,21]. At the same time, more and more solid wastes and new materials are also being used in concrete materials and structures [22]. The relevant research aims to improve our understanding of the performance and environmental impact of concrete materials and structures in a freeze–thaw environment, which is very important for sustainable building practices. Wei et al. discussed the in situ and structural application of waste concrete in cold areas and comprehensively evaluated its sustainable application potential in freeze–thaw environments from three aspects: performance, environmental load, and economic benefits based on energy analysis [23]. Manawadu et al. studied the freeze–thaw durability of the bond in shotcrete between the new and old concrete and the concrete interface under tensile conditions [24]. However, the mechanism behind this relationship remains elusive.

Another line of research has focused on the mechanisms responsible for freeze–thaw damage in concrete. One commonly accepted mechanism is the stress concentration around inclusions due to the volume expansion of ice during freezing [25,26]. This stress concentration leads to the formation of microcracks, which then propagate and coalesce during subsequent freeze–thaw cycles [27,28]. However, recent studies have also investigated other potential mechanisms such as chemical reactions and material defects [29,30,31]. Moreover, research has also focused on methods to improve the freeze–thaw resistance of concrete [32,33]. These methods include using modified cementitious materials [34], optimizing the mix design [35], using anti-freeze agents [36], and enhancing the durability of the cementitious matrix. Gołaszewski et al. discussed the influence of adding limestone powder on freeze–thaw resistance, which would be beneficial for the frost resistance of concrete [37]. These methods have shown varying degrees of success in improving the freeze–thaw resistance of concrete, but further research is needed to identify more effective solutions. Overall, previous research has provided insights into the freeze–thaw damage characteristics of concrete. For the discussed approaches to freeze–thaw damage, this study focuses on the damage mechanism of water expansion. However, there are still gaps in our understanding of the relationship between mechanical properties and freeze–thaw damage as well as the mechanisms responsible for this damage. Therefore, further research is needed to improve our understanding of these characteristics and develop more effective methods to mitigate freeze–thaw damage in concrete.

Thus, in order to explore mechanical performance and deterioration under freeze–thaw conditions, taking a common mix proportion of concrete as the research object, this paper investigates freeze–thaw damage characteristics for concrete through its compressive mechanical performance as well as acoustic parameters. This study aims to determine the relationship between these properties and the damage caused by freeze–thaw cycling. It also seeks to identify the mechanisms responsible for this damage and provide insights into the damage evolution process. These insights into freeze–thaw damage characteristics based on mechanical properties and acoustic parameters can support efforts to enhance the performance and durability of structures in cold regions or under similar conditions.

## 2. Materials and Methods

### 2.1. Experimental Materials and Mixing Ratio

In this study, ordinary Portland cement (P.O 42.5) from Jilin Yatai Cement Co., Ltd. (Changchun, China), was used for the cementitious materials, and fly ash was also used as one of the binder materials. Manufactured sand with a fineness modulus of 2.6 was used as the fine aggregate. Ordinary gravel with a continuous gradation of 5~16 mm diameter was used as the coarse aggregate. Regular tap water was used. The research object is a kind of concrete commonly used in engineering, and its mix proportion is given in Table 1.

**Figure 1:**
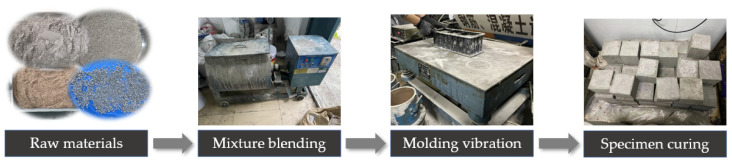
The concrete specimen preparation flow, based on the dry-mixing method.

Following the Chinese specification “Standard for test methods of concrete physical and mechanical properties” (GB/T 50081-2019), all concrete specimens had cube dimensions of 100 mm × 100 mm × 100 mm. In this experiment, the dry-mixing method was used for concrete mixing, and the specimen preparation flow, such as the order of adding raw materials and the mixing process, was carried out according to the specimen preparation flow in Figure 1. The experimental concretes were made strictly according to the specimen preparation process, and then transferred to the standard curing room after demolding. The curing room conditions included a relative humidity of 95%, and the temperature was kept at 20 °C ± 2 °C. Then, after curing for 28 days, the subsequent tests could be carried out for these cured specimens.

### 2.2. Experimental Procedure

**Figure 2:**
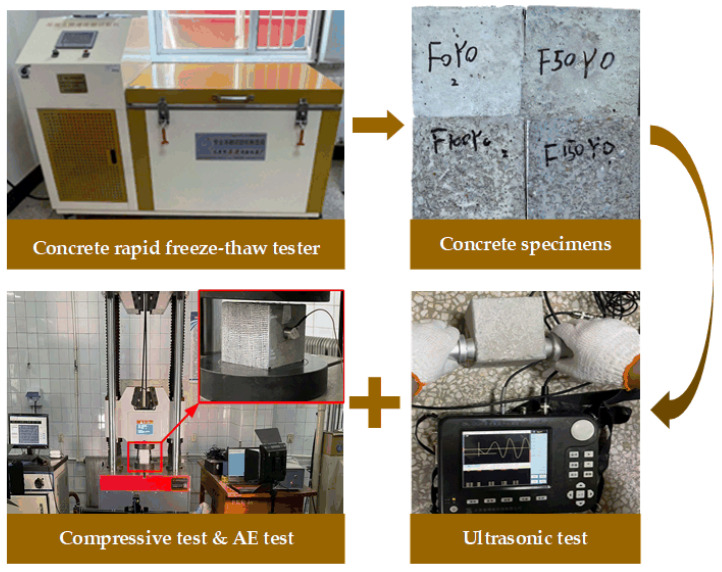
Experimental procedure flow for the study of the durability of the concretes.

The experimental procedure flow for the study of the durability of concrete materials exposed to the freezing–thawing effect is in Figure 2. The experiment design consisted of four groups, separated according to the number of freeze–thaw cycles they underwent: group Nos. 1–4 represented concrete with 0, 50, 100, and 150 freeze–thaw cycles, respectively. After specimen preparation and the curing process, the concrete specimens underwent freeze–thaw cycle testing, which was conducted using a rapid freeze–thaw environment experimental chamber. Each group contained 3 samples, and mean values of the experimental results for these three specimens were taken for analysis of their compressive mechanical properties and acoustic parameters.

#### 2.2.1. Freeze–Thaw Cycle Test

In this study, the freeze–thaw cycle test adopted a concrete rapid freeze–thaw tester to control the temperature. Based on the Chinese standard (GB/T 50082-2009) [38], the lowest and highest temperatures in the center should be set to (−18 ± 2) °C and (5 ± 2) °C, respectively. Each freeze–thaw cycle should be completed within 2~4 h, and the time for melting should not be less than 1/4 of the whole freeze–thaw cycle time. The conversion time between freezing and thawing should not exceed 10 min. Concrete specimens were removed from the curing chamber under a constant temperature and humidity, and the initial weight, length, and height of each specimen were recorded. We placed the specimens in a freezing and thawing device that met the requirements of the test specification. This process was repeated for a certain number of times according to the requirements of the test specification. After a certain amount of freezing and thawing cycles, each specimen was removed from the freezing and thawing device, followed by measuring its weight, length, and height, and recording these data as well as monitoring the mass loss. Meanwhile, we needed to observe whether there were any significant changes in appearance or cracking in the specimens after each certain freezing and thawing cycle.

#### 2.2.2. Compressive Mechanical Test

The compressive mechanical properties test was carried out with reference to the requirements of Chinese specification (GB/T 50081-2019) [39]. The experimental specimens were removed from the constant temperature and humidity chamber or the rapid freeze–thaw environment test chamber and we recorded the initial weight, length, and height of each specimen. We placed the specimens in a compressive strength testing machine that met the requirements of the test specification directly. Then, at room temperature, we started the microcomputer-controlled electronic universal testing machine with a maximum force of 1000 kN and compressed the specimens at a constant load rate of 0.3 kN/s until failure. Finally, the compressive strength data were recorded and analyzed to calculate the compressive strength of the concrete, evaluating its compressive strength performance.

#### 2.2.3. Acoustic Detection Test

Ultrasonic testing was selected as a non-destructive experimental method. Based on the influence of internal defects, pores, cracks and other heterogeneity, when ultrasonic waves propagate in the material being tested, it can result in different acoustic characteristics, which can be used to assess the internal quality and structural status of concrete. In this study, a U520 non-metallic ultrasonic tester from Beijing ZBL Science and Technology Co., Ltd. (Beijing, China) was used to measure the change in the internal ultrasonic propagation velocity of the specimens under different freeze–thaw cycles.

Acoustic emission (AE) technology is a sensitive passive monitoring method. By capturing and analyzing the AE signals generated in the mechanical process, information on internal damage changes can be obtained indirectly. In this study, the acoustic emission signals of the compression failure process of specimens in freeze–thaw cycles were collected to explore their compression failure damage characteristics. An AE testing system from Qingcheng AE Institute Co., Ltd. (Guangzhou, China) was used as the acoustic system with one S150-type acoustic sensor, for which the used threshold of the acoustic signals was 40 dB. While carrying out the mechanical test, the AE test needed to be carried out simultaneously to achieve synchronization of the experimental data obtained from the loading frame and the acoustic system.

## 3. Results and Discussion

### 3.1. Freeze–Thaw Damage of Concrete Based on Macro Morphology and Compressive Mechanical Properties

#### 3.1.1. Mass Loss

**Figure 3:**
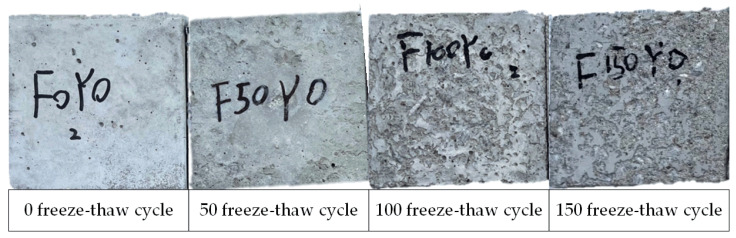
The apparent morphology of concrete with increasing freeze–thaw cycles.

The apparent morphology of the concrete specimens after undergoing different degrees of freeze–thaw cycles is shown in Figure 3. The concrete specimens have a smooth surface and intact appearance during the non-freezing–thawing stage. After experiencing 50 freeze–thaw cycles, the apparent morphology of the concrete specimens showed almost no significant change, but some dense pores could be observed on the specimen surface. As the freezing–thawing damage increased, the degree of concrete specimen surface damage gradually intensified. After experiencing 100 freeze–thaw cycles, there was a significant peeling of cement mortar on the concrete specimen surface. After 150 freeze–thaw cycles, the cement mortar on the surface peeled off severely, exposing a significant area of coarse aggregates.

**Figure 4:**
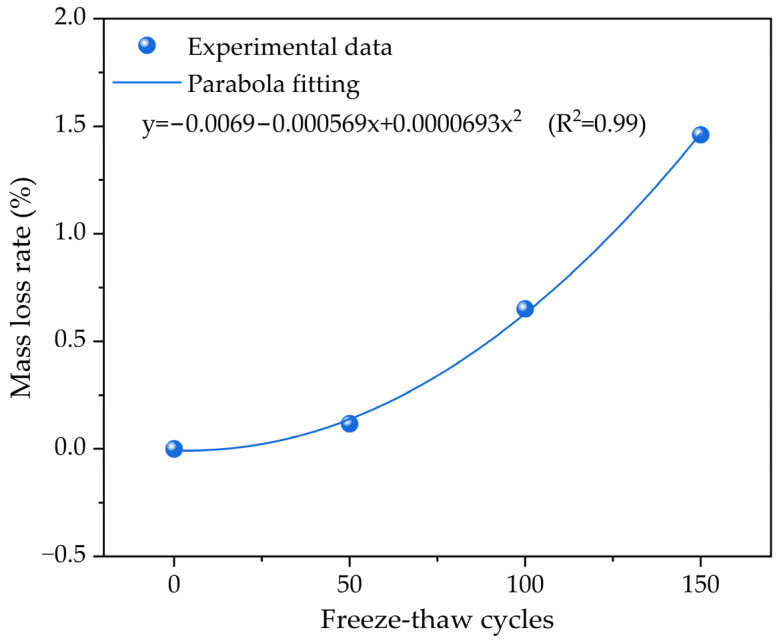
The relationship of the cumulative mass loss of the concrete with the number of freeze–thaw cycles.

Figure 4 illustrates the correlation between cumulative mass loss and the number of freeze–thaw cycles. The results indicate that as the number of freeze–thaw cycles increases from 0 to 150, the correlation curve between the cumulative mass loss and freeze–thaw cycles shows a gradually increasing trend. When the number of freezing–thawing cycles is 50, the cumulative mass loss reaches approximately 0.12%. As the freezing–thawing cycles increase, the cumulative mass loss increases at a faster rate. When the freezing–thawing cycles reach 100 times, the cumulative mass loss increases to approximately 0.65%, and the cumulative mass loss increases to approximately 1.46% at 150 freeze–thaw cycles. Especially worth noting is that after 50 freezing–thawing cycles, the slope of the cumulative mass loss curve is significantly increased. As the freezing–thawing damage degree to the concrete specimen increases, the cumulative mass loss curve becomes steeper, indicating a faster rate of change in the concrete mass. The fitting results of the relationship between the cumulative mass loss of concrete and the freeze–thaw cycles during the freeze–thaw cycling process reveal that they follow a parabola fitting pattern. As the number of freeze–thaw cycles increases, the cumulative mass loss gradually increases and the apparent morphology becomes more defective. These structural defects mainly include microcracks and debonding between the aggregates and cement paste. This is mainly due to water content in the concrete expanding during freezing and contracting during thawing, resulting in microcracks and debonding in the concrete structure. These structural defects can significantly reduce its mechanical properties and service life. Therefore, it is necessary to take measures to reduce the water content or improve its freeze-resistant ability during concrete construction and use to ensure its service life.

#### 3.1.2. Compressive Mechanical Properties

**Figure 5:**
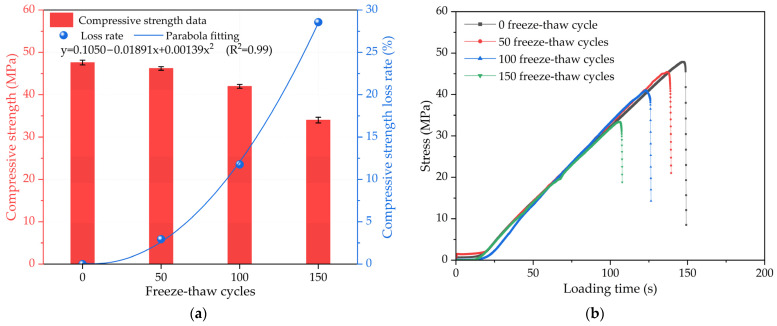
The compressive mechanical properties: (**a**) relationship between compressive strength of concrete and the number of freeze–thaw cycles; (**b**) stress–time curves under different amounts of freeze–thaw cycles.

The typical relationship between the compressive strength of the concrete and the number of freeze–thaw cycles is plotted in Figure 5a. Generally speaking, the compressive strength of the concrete decreases with the increase in freezing–thawing cycles. During freeze–thaw cycling, the compressive strength of concrete is also affected by various factors such as water content, temperature, and the presence of freezing-sensitive materials. After 150 freeze–thaw cycles, the strength of the concrete decreased from 47.6 MPa to approximately 34 MPa. However, this trend is not linear. The decreased rate of compressive strength may increase at a faster rate as the number of freeze–thaw cycles increases. The loss rate results of compressive strength show that the compressive strength of concrete gradually decreases with the increase in freezing–thawing cycles. When the number of freeze–thaw cycles is 50, the compressive strength loss rate reaches approximately 2.94%. As the number of freeze–thaw cycles increases, the compressive strength loss rate increases at a faster rate. When the number of freeze–thaw cycles reaches 100, the compressive strength loss rate increases to approximately 11.76%. The compressive strength loss rate even increases to approximately 28.57% after 150 freeze–thaw cycles. The fitting results of the relationship between the strength loss rate and the freeze–thaw cycles reveal that they also follow a parabola fitting pattern, which is similar to the cumulative mass loss. As the degree of freezing–thawing damage to the concrete specimen increases, the compressive strength loss rate curve becomes steeper, indicating a faster rate of change in concrete strength. Freeze–thaw cycling has a major effect on strength. The concrete has less damage in the initial stage and a denser internal pore structure. This is mainly due to water content in the concrete expanding during freezing and contracting during thawing, resulting in microcracks and debonding in the concrete structure. These small damages can lead to structural defects, which can significantly reduce the mechanical properties and service life of concrete. Figure 5b shows the stress–time curves of the concretes under different freeze–thaw cycles. It can be seen that the failure stress of the concretes decreases with the freeze–thaw cycle, and the corresponding loading time is also shortened.

### 3.2. Acoustic Parameters of Concrete under Freeze–Thaw Cycles

The characterization of acoustic parameters such as the ultrasonic wave velocity and acoustic emission signals has a great impact on the performance of concrete. The propagation velocity of ultrasonic waves is a crucial physical parameter that is closely related to the material density, elastic modulus, and other microstructural properties. The energy and count of acoustic emission signals are mainly determined by the strength and damage of the concrete. In short, acoustic parameters have significant significance in evaluating the performance of concrete during freezing and thawing processes.

#### 3.2.1. Ultrasonic Pulse Velocity Loss Rate

**Figure 6:**
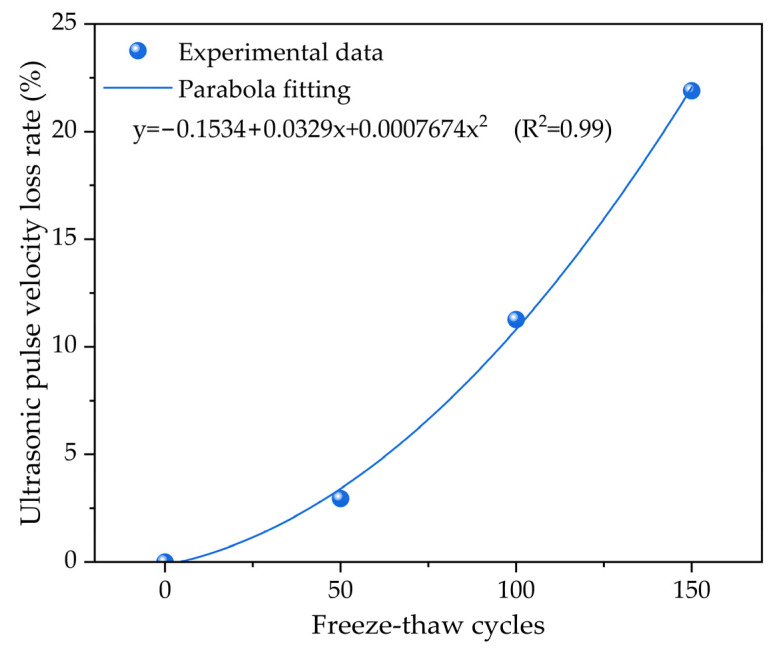
The relationship of the ultrasonic pulse velocity loss rate of the concrete with the number of freeze–thaw cycles.

Figure 6 presents the relationship of the ultrasonic pulse velocity loss rate of the concrete versus the freeze–thaw cycles. During freeze–thaw cycling, the ultrasonic wave velocity of concrete also changes with the number of freezing–thawing cycles. Generally speaking, as the number of freeze–thaw cycles increases, the ultrasonic wave velocity for the concrete decreases. This trend can be explained by the principle of ultrasonic wave propagation in concrete. Ultrasonic waves propagate in concrete through the interaction between the elastic stress field and the density field. When an ultrasonic wave propagates in concrete, it generates a compressive stress field in the material, leading to a density field distribution. The propagation velocity of ultrasonic waves is determined by the elastic modulus as well as the density. In freeze–thaw cycling, repeated freezing and thawing causes microcracks and defects in the concrete structure, which reduces the elastic modulus as well as the density and ultimately leads to a decrease in the ultrasonic wave velocity.

The results show that ultrasonic wave velocity gradually decreases with the freezing–thawing cycles. When the number of freeze–thaw cycles is 50, the ultrasonic wave velocity loss rate reaches approximately 2.95%. As the freeze–thaw cycles increase, the ultrasonic wave velocity loss rate increases at a faster rate. When the number of freeze–thaw cycles reaches 100, the ultrasonic wave velocity loss rate increases to approximately 11.27%, while the ultrasonic wave velocity loss rate increases to approximately 21.89% after 150 freeze–thaw cycles. The above results indicate that freeze–thaw cycling has a significant effect on the ultrasonic wave velocity of concrete. With respect to the polynomial fitting analysis of the ultrasonic wave velocity loss rate, it can be fitted using a quadratic polynomial equation. As the number of freeze–thaw cycles increases, the ultrasonic wave velocity loss rate shows a parabola trend. This trend may be attributed to the fact that as freeze–thaw cycling progresses, microcracks and defects in the concrete gradually increase, resulting in a rapid decrease in the ultrasonic wave velocity loss rate after a certain amount of freeze–thaw cycles. In summary, this finding provides valuable insights into monitoring and predicting the mechanical properties and service life of concrete in freeze–thaw cycling.

#### 3.2.2. Analysis of AE Rate of Concrete during Compressive Failure Process

**Figure 7:**
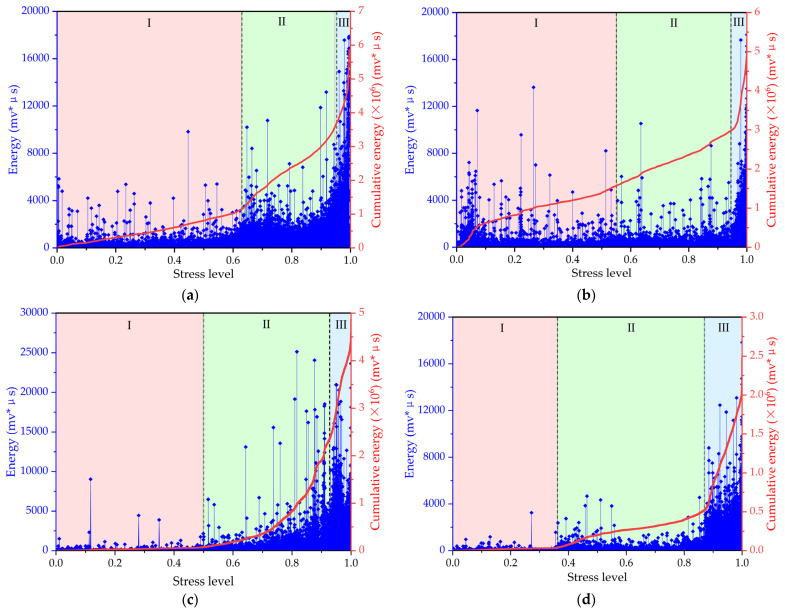
The relationship of the AE energy parameters of the concrete with the stress level under freeze–thaw cycling: (**a**) 0 freeze–thaw cycles; (**b**) 50 freeze–thaw cycles; (**c**) 100 freeze–thaw cycles; (**d**) 150 freeze–thaw cycles.

Figure 7 illustrates the correlation between the AE energy parameters and the stress level with freezing–thawing cycles. As shown in Figure 7, according to the value level as well as the cumulative value level of the AE parameter, the entire failure and cracking process of concrete under compressive loads can be divided into three stages, i.e., the linear growth stage, damage accumulation stage, and failure stage.

Stage I—linear growth stage: When the load is less than 40~60% of the peak load, the stress–AE cumulative energy curve of the concrete shows linear growth behavior, and the AE cumulative energy increases with the increase in load. The first stage is the stage of crack generation for concrete microdamage. When the compressive load is applied to the concrete specimen, microcracks begin to appear inside the concrete specimen as the load level increases. Due to the small degree of damage to the specimens in the early stage of compression, concrete specimens have sufficient strength to resist compression, resulting in lower values of energy than in other stages. For the specimens subjected to 50 freeze–thaw cycles, there are some energy peaks or count peaks in the early stage, which may be caused by the uneven surface of the specimen and the noise of the acoustic emission sensor.

Stage II—damage accumulation stage: When the load is between 40% and 90% of the peak load, the stress–AE cumulative energy curve shows nonlinear growth behavior, and the AE cumulative energy increases slowly with the increase in load. The second stage is the stage of concrete cracking, damage accumulation, and expansion. At this stage, the internal microcracks in the concrete will gradually increase and expand, and a certain amount of plastic deformation will occur. The energy value recorded with AE technology also increases at a faster rate.

Stage III—failure stage: While the load reaches about 85~90% of the peak load, the stress–AE cumulative energy curve shows a sharp increase, indicating that the concrete is approaching failure. At this stage, the concrete specimen gradually reaches its maximum bearing capacity. The internal microcracks in the concrete will expand rapidly, and a large number of cracks will appear on the surface of the concrete. The AE signal recorded with the AE system reaches its peak, and the values of AE energy on the left vertical axes reach the maximum, approximately close to 20,000 mv*μs. At this moment, the specimen is obviously fractured, and the compressive bearing capacity of the specimen is lost.

**Figure 8:**
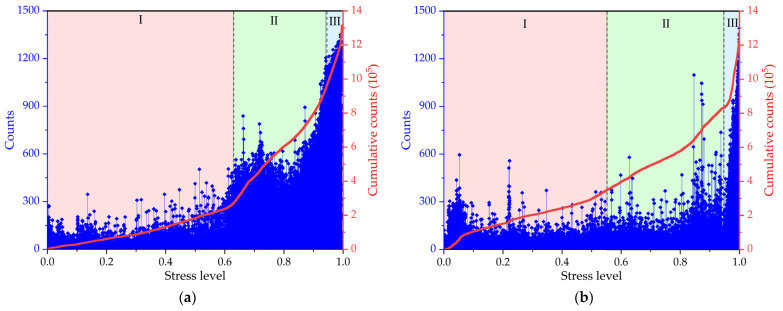

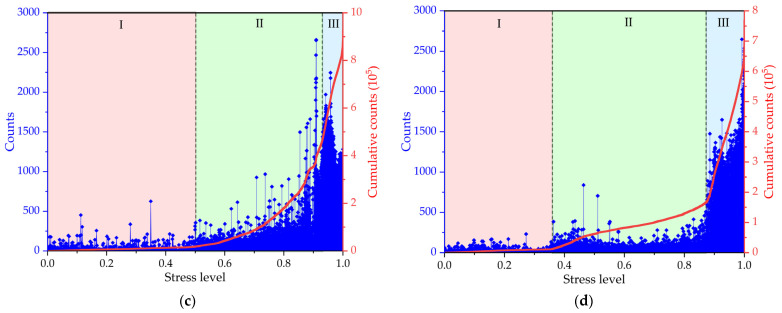
The relationship of the AE count parameters of the concrete with the stress level under freeze–thaw cycling: (**a**) 0 freeze–thaw cycles; (**b**) 50 freeze–thaw cycles; (**c**) 100 freeze–thaw cycles; (**d**) 150 freeze–thaw cycles.

Figure 8 illustrates the correlation between the AE count parameters and the stress level. Under the same freezing–thawing action, the energy–stress level and AE count–stress level curves of the specimen exhibit similar evolution patterns during the compression failure process. And the entire failure process of concrete under compressive loads in relation to the AE count can be divided into the aforementioned three stages, i.e., the linear growth stage, the damage accumulation stage, and the failure stage. Stage I—linear growth stage: When the load is less than 40~60% of the peak load, the stress–AE cumulative count curve of the concrete shows linear growth behavior, and the AE cumulative count increases with the increase in load. The first stage is the stage of microcrack generation inside the concrete, in which the concrete specimens have sufficient strength to resist compression, resulting in a lower AE count [40]. Stage II—damage accumulation stage: When the load is between 40% and 90% of the peak load, the stress–AE cumulative count curve shows nonlinear behavior, and the AE cumulative count increases slowly with the increase in load. The second stage is the stage of concrete cracking and expansion, in which the internal microcracks of the concrete will gradually increase and expand with damage accumulation, and the count value collected with the AE technology also increases at a faster rate. Stage III—failure stage: When the load reaches about 85~90% of the peak load, the stress–AE cumulative count curve shows a sharp increase, indicating that the concrete is approaching failure. At this stage, the concrete specimen gradually reaches its maximum bearing capacity. The internal microcracks in the concrete will expand rapidly, and a large number of cracks will appear on the surface of the concrete. The values of AE count on the left vertical axes reach their maximum, approximately close to or above 1500 counts, showing that the specimen has an obvious fracture. The compressive bearing capacity of the specimen is then lost. The internal structure of concrete material is complex, which may affect the amplitude and distribution of AE signals. What is interesting in Figure 7c and Figure 8c is that there is a drop in the AE energy or count at about a 92% stress level, which is different from the other Figure 8a,b,d. This finding could be due to concrete microcracks accumulating and producing concentrated stress in the compression process, which might make the AE signal drop at a larger value level in the later failure stage.

From the relationship between the AE parameters (energy, count) and the stress level under freezing–thawing cycles, it can be seen that the period of stage I, the linear growth stage, shortens with increases in the freeze–thaw cycles. This is because the freezing–thawing process causes damage to the internal microstructure of concrete, resulting in a decrease in its elastic modulus and an increase in its internal defects. Therefore, the linear growth stage shortens with increases in the freeze–thaw cycles. As for stage II, the damage accumulation stage, this stage period increases with the increase in freeze–thaw cycles. This is because the freezing–thawing process gradually degrades the material properties, and the plastic deformation becomes more significant as the damage accumulates. What is interesting about the relationship in the figures is that the period of stage III—the failure stage—appears earlier with increases in the number of freeze–thaw cycles, which could be attributed to internal defects and cracks expanding rapidly under the freezing–thawing action.

In short, the freezing–thawing process has an obvious effect on the acoustic emission parameters of concrete. The linear growth stage shortens, the damage accumulation stage increases, and the failure stage appears earlier with increases in the number of freezing–thawing cycles. These changes can be used to evaluate the damage status and performance degradation of concrete during freezing–thawing action.

## 4. Conclusions

In order to explore its mechanical performance and deterioration in freezing–thawing conditions, this work focused on evaluating the compressive mechanical properties and acoustic parameters of concrete. A compressive mechanical test, mass loss analysis, and ultrasonic pulse velocity test were applied to evaluate the freezing–thawing damage characteristics of concrete. After that, AE technology was used to assess the damage development status of the concrete. The study’s findings are summarized as follows:

(1) The relationship between the cumulative mass loss of concrete and the freeze–thaw cycles during the freeze–thaw process follows a parabola fitting pattern. As the freezing–thawing damage degree of the concrete specimens increases, the surface of the concrete specimens presents a trend of “smooth intact surface” to “surface with dense pores” to “cement mortar peeling” to “coarse aggregates exposed on a large area”.
(2) The compressive mechanical test revealed that freezing–thawing conditions have a considerable impact on concrete. The relationship between the loss rate of compressive strength and the freeze–thaw cycles also follows a parabola fitting pattern, which is similar to the cumulative mass loss. As the number of freeze–thaw cycles increases, the compressive strength loss rate increases at a faster rate.
(3) The ultrasonic test findings indicated that as the number of freeze–thaw cycles increases, the ultrasonic wave velocity loss rate shows a parabola trend. This trend may be attributed to the fact that as freeze–thaw cycling progresses, microcracks and defects in the concrete gradually increase, resulting in a rapid decrease in the ultrasonic wave velocity loss rate after a certain number of freeze–thaw cycles.
(4) Based on the value level as well as the cumulative value level of the AE parameter, the entire compressive failure process can be divided into three stages. The stress–AE cumulative parameter curve shows linear growth behavior in the linear growth stage with a load of less than 40~60%. The stress–AE cumulative parameter curve shows nonlinear growth behavior in the damage accumulation stage with a load between 40% and 90%. After that, the stress–AE cumulative parameter curve shows a sharp increase in the failure stage.
(5) The linear growth stage shortens, the damage accumulation stage increases, and the failure stage appears earlier with the increase in the number of freeze–thaw cycles. These changes can be used to evaluate the damage status and performance degradation of concrete during freezing–thawing action.

The innovation of this study lies in providing a comprehensive understanding of the freeze–thaw damage characteristics of concrete based on compressive mechanical properties and acoustic parameters. The results can be used to evaluate the performance degradation and damage status of concrete under freeze–thaw conditions and guide the repair and maintenance work of relevant structures. In further work, the influence of concrete mix proportions and ingredient types should be analyzed, and freeze–thaw-resistant materials suitable for concrete should be developed accordingly.

## Figures and Tables

**Table 1:** The mix proportion of the concrete used (unit: kg/m^3^).

Water–Binder Ratio	Water	Cement	Fly Ash	Medium Sand	Ordinary Gravel
0.44	210	430	47	565	1148

## Data Availability

Data are contained within the article.
